# TAK1 inhibitor 5Z-7-oxozeaenol sensitizes cervical cancer to doxorubicin-induced apoptosis

**DOI:** 10.18632/oncotarget.16895

**Published:** 2017-04-06

**Authors:** Shan Guan, Jiaxiong Lu, Yanling Zhao, Sarah E. Woodfield, Huiyuan Zhang, Xin Xu, Yang Yu, Jing Zhao, Shayahati Bieerkehazhi, Haoqian Liang, Jianhua Yang, Fuchun Zhang, Surong Sun

**Affiliations:** ^1^ Xinjiang Key Laboratory of Biological Resources and Genetic Engineering, College of Life Science and Technology, Xinjiang University, Urumqi, Xinjiang 830046, China; ^2^ Texas Children's Cancer Center, Department of Pediatrics, Dan L. Duncan Cancer Center, Baylor College of Medicine, Houston, TX 77030, USA; ^3^ Division of Pediatric Surgery, Michael E. DeBakey Department of Surgery, Dan L. Duncan Cancer Center, Baylor College of Medicine, Houston, TX 77030, USA; ^4^ Department of Labour Hygiene and Sanitary Science, College of Public Health, Xinjiang Medical University, Urumqi, Xinjiang 830011, China; ^5^ School of Pharmacy, Zhengzhou University, Zhengzhou, Henan 450001, China

**Keywords:** cervical cancer, TAK1 inhibitor, 5Z-7-oxozeaenol, chemotherapy, doxorubicin

## Abstract

Aberrant activation of nuclear factor-κB (NF-κB) allows cancer cells to escape chemotherapy-induced cell death and acts as one of the major mechanisms of acquired chemoresistance in cervical cancer. TAK1, a crucial mediator that upregulates NF-κB activation in response to cellular genotoxic stress, is required for tumor cell viability and survival. Herein, we examined whether TAK1 inhibition is a potential therapeutic strategy for treating cervical cancer. We found that TAK1 inhibitor 5Z-7-oxozeaenol significantly augmented the cytotoxic effects of Dox in a panel of cervical cancer cell lines. Treatment with 5Z-7-oxozeaenol hindered Dox-induced NF-κB activation and promoted Dox-induced apoptosis in cervical cancer cells. Moreover, 5Z-7-oxozeaenol showed similar effects in both positive and negative human papillomavirus-infected cervical cancer cells. Taken together, our results provide evidence that TAK1 inhibition significantly sensitizes cervical cancer cells to chemotherapy-induced cell death and supports the use of TAK1 inhibitor with current chemotherapies in the clinic for patients with refractory cervical cancer.

## INTRODUCTION

As the second leading cause of cancer-related deaths in women, cervical cancer is a major health problem with an evergrowing threat worldwide [1–3]. Despite that, more than 80% of early period and 60% of loco-regionally advanced cervical cancers exhibit good outcomes with current treatment regimens [4]. However, due to resistance mechanisms, the effects of chemotherapeutic and radiotherapeutic agents, such as the traditional chemotherapies doxorubicin (Dox) and etoposide (VP-16), are thereby reduced [5–7].

Transcription factor nuclear factor-κB (NF-κB), known as one of the critical mediators of diverse functions in cancer cells, is involved in inflammatory cellular responses to stimuli such as stress, cytokines, free radicals, ultraviolet irradiation, oxidized low-density-lipoprotein (LDL), and bacterial and viral antigens [8–10]. Aberrant activation of NF-κB is frequently seen in tumor cells, including cervical cancer cells, and is believed to be one of the reasons for the development of resistance mechanisms that contribute to resistance to current chemo- and radiotherapies [11–13]. In cervical carcinoma, the majority of chemotherapies, radiotherapies, and combinations of chemo- and radiotherapies activate the NF-κB signaling pathway, which results in abnormally increased NF-κB expressionand, in turn, promotes the development of refractory disease [14].

TAK1, one of the mitogen-activated protein kinase (MAPK) family members, is reported as a pivotal intermediate for activation of transforming growth factor-β (TGF-β), IκB kinase (IKK), or MAPK, as well as a critical player in innate and adaptive immune responses [15–20]. In addition, it has been widely proven to be a crucial mediator of upregulation of NF-κB activation in response to genotoxic stress or Dox exposure [21–25]. Accordingly, NF-κB activation accelerates chemoresistance induced by conventional chemotherapies and promotes tumor cell viability. Thus, inhibition of TAK1 blocks NF-κB activation, resulting in the death of more vulnerable cancer cells in studies of colon cancer [26], leukemia [27, 28], cerebral ischemia [29], and cervical cancer [4]. Therefore, TAK1 inhibition to inactivate the NF-κB pathway is a promising therapeutic strategy [30–32].

5Z-7-oxozeaenol (5Z-7) is a novel TAK1 inhibitor that significantly augments the cytotoxic effects of Dox and VP-16 [33]. 5Z-7 efficiently overcomes chemoresistance induced by NF-κB activation [33–35] and exhibits strong efficacy in studies of breast cancer [36], neuroblastoma [37], and lymphoma [38]. However, the effects of 5Z-7 in cervical cancer remains unknown.

In this study, we evaluate the therapeutic effects of the TAK1 inhibitor 5Z-7 on cervical cancer. Our results demonstrate that 5Z-7 significantly augments the cytotoxic effects of Dox in a panel of cervical cancer cell lines. Treatment with 5Z-7 enhances the anti-proliferative effects of Dox in an anchorage-independent manner, hinders Dox-induced NF-κB activation, and increases Dox-induced apoptosis. Interestingly, 5Z-7 showed similar effect in both positive and negative human papillomavirus (HPV)-infected cervical cancer cells. Importantly, our preclinical study of 5Z-7 supports further studies of TAK1 inhibitor and its potential use in the clinic for patients with refractory cervical cancer.

## RESULTS

### TAK1 inhibitor 5Z-7-oxozeaenol exhibits cytotoxic effect on cervical cancer cells

**Figure 1:**
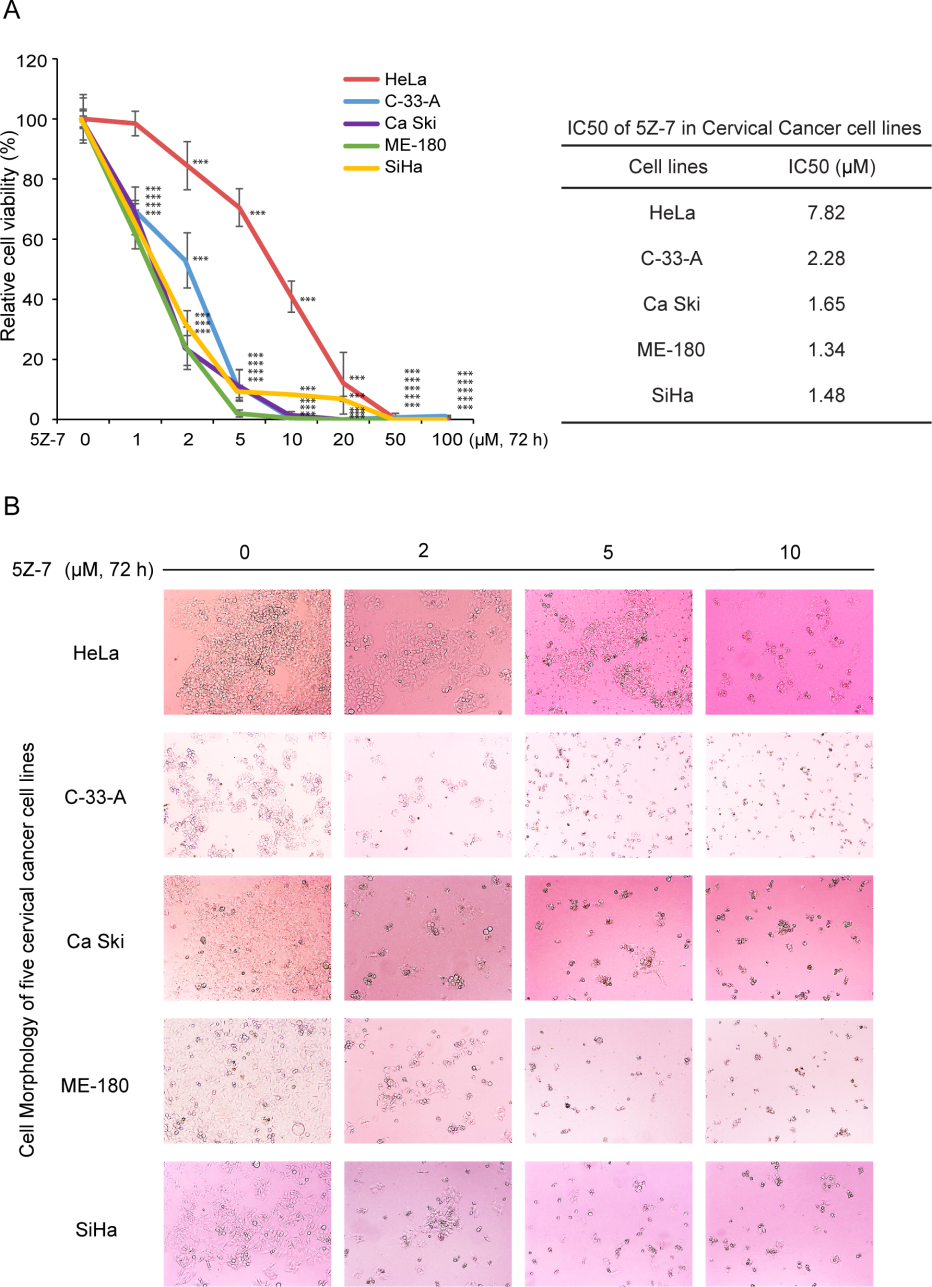
5Z-7-oxozeaenol suppressescell proliferation of cervical cancer cells (**A**) Five cervical cancer cell lines were seeded into 96-well plates at a concentration of 1 × 10^4^ perwell and treated with indicated concentrations of 5Z-7 or DMSO. Cell viability was assessed by MTT assays after 72 h of exposure to drug. Data was represented as the mean standard deviation (SD) with *P* < 0.05 (*), *P* < 0.01 (**), or *P* < 0.001 (***) (ANOVA) as indicated. IC50 values of 5Z-7 in cervical cancer cell lines were listed. (**B**) Morphological changes of five different cervical cancer cell lines treated with increasing concentrations (0 μM, 2 μM, 5 μM, 10 μM) of 5Z-7 were shown.

To determine the inhibitory effects of 5Z-7 on cervical cancer cell lines, we conducted the MTT assay on five cervical cancer cell lines, including HeLa, C-33-A, Ca Ski, ME-180 and SiHa (Figure 1A). The IC50s of 5Z-7 on these cell lines were calculated and ranked from 1.34 μM to 7.82 μM (Figure 1A). The results exhibited that TAK1 inhibitor 5Z-7 could inhibit cervical cancer cell proliferation (Figure 1A, 1B).

### TAK1 inhibitor 5Z-7-oxozeaenol significantly increases the cytotoxic effect of Dox on cervical cancer cells

**Figure 2:**
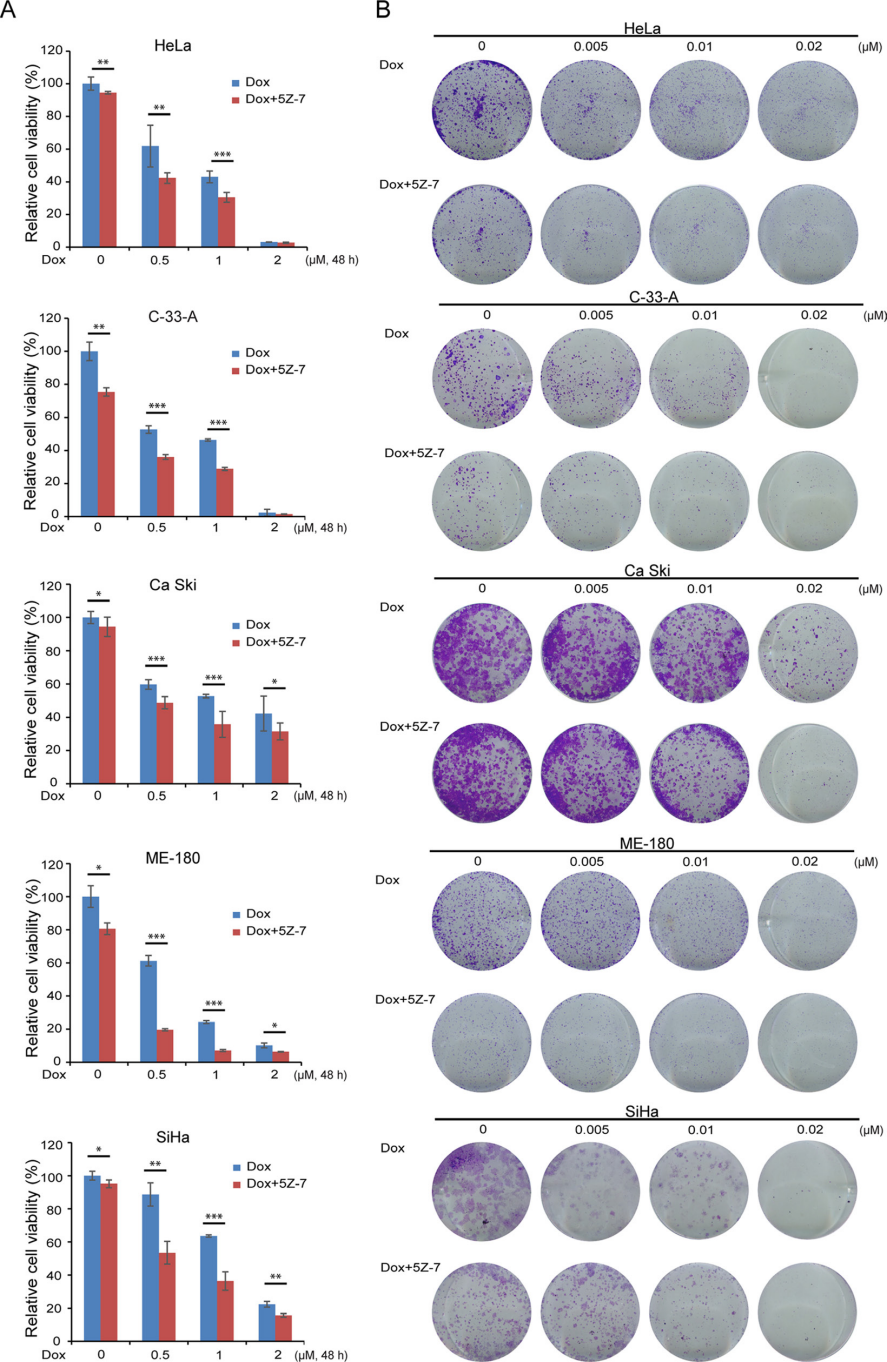
5Z-7-oxozeaenol enhances Dox-induced growth inhibitory effect on cervical cancer cells (**A)** HeLa, C-33-A, Ca Ski, ME-180 and SiHa were treated with Dox at the indicated concentrations with or without 2 μM 5Z-7 for 48 h. The cell viability was then measured by CCK-8 assay. All values are expressed as the mean standard deviation (SD). *P* values < 0.05 (*), < 0.01 (**), or < 0.001 (***) were indicated. (**B)** Five cervical cancer cell lines wereseeded in 12-well plates at 2 × 10^3^ per well, and then incubated with Dox at the indicated concentrations with or without 2 μM 5Z-7 for 72 h. The cell colonies were fixed, stained and photographed.

To identify whether TAK1 inhibitor 5Z-7 could inhibit NF-κB pathway and thereby block NF-κB activation-induced chemoresistance, we treated a panel of five cervical cancer cell lines, including HeLa, C-33-A, Ca Ski, ME-180 and SiHa, with Dox, a NF-κB activator, and the TAK1 inhibitor 5Z-7 for 48 h (Figure 2A). To uncover the combination effect of 5Z-7 and Dox on cell proliferation, we performed colony formation assay to detect anti-proliferation effects of 5Z-7 and Dox combinations (Figure 2B). The results of both CCK-8 and colony formation assays with these cells demonstrate that TAK1 inhibition significantly enhances the cytotoxic effect of Dox on cervical cancer cells, as compared to the cells treated with either single agent. In addition, the efficacy of 5Z-7 is independent of HPV virus status, since both HPV positive (HeLa, Ca Ski, ME-180, SiHa) and HPV negative (C-33-A) cervical cell lines showed similar results.

### TAK1 inhibitor 5Z-7-oxozeaenol strengthens the Dox-mediated inhibitory effects on anchorage-independent growth of cervical cancer cells

**Figure 3:**
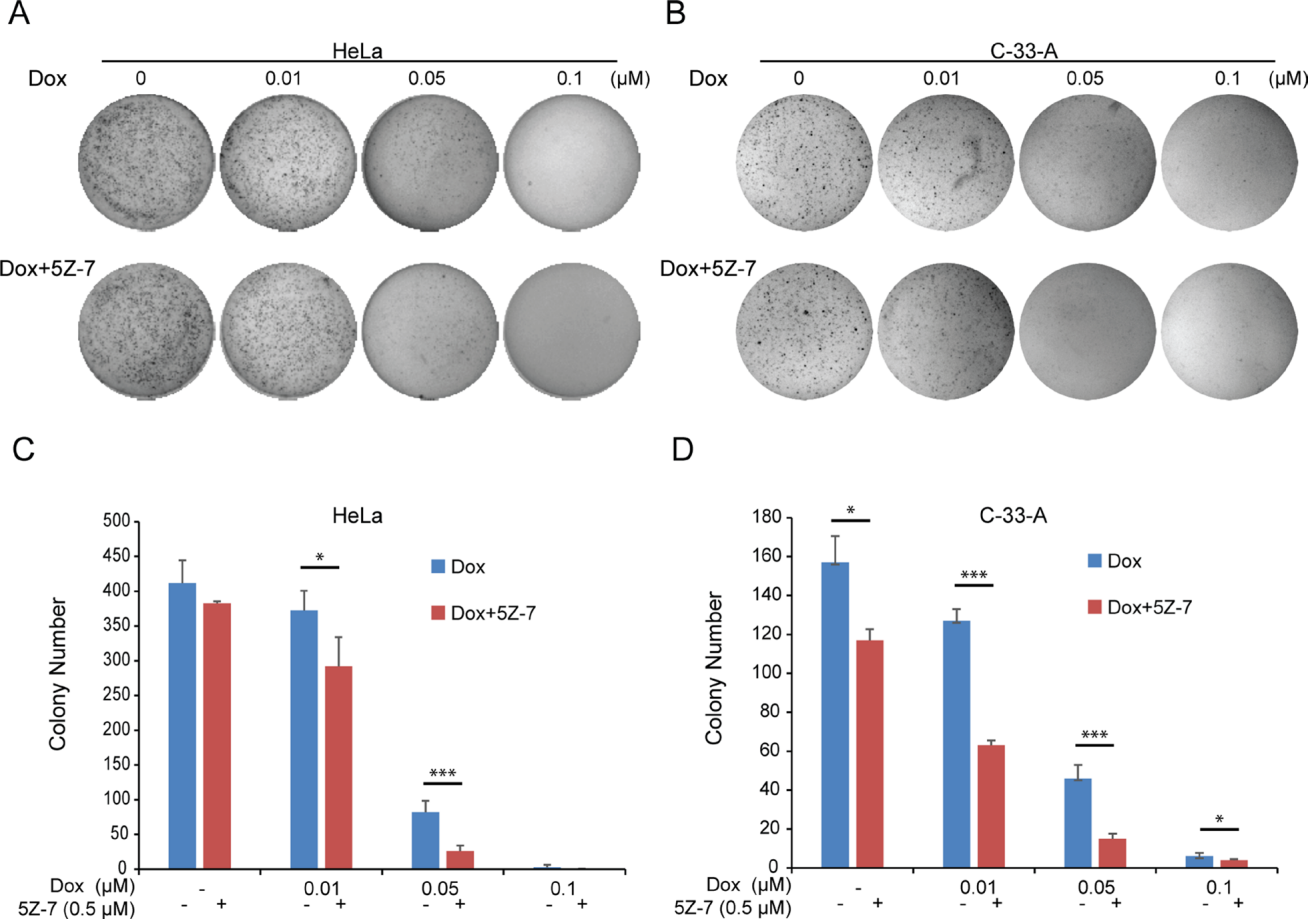
5Z-7-oxozeaenol inhibits anchorage-independent growth on two cervical cancer cells (**A**–**B)** Soft agar assays of HeLa (A) and C-33-A (B) cells treating with increasing concentrations (0 μM, 0.01 μM, 0.05 μM, 0.1 μM) of Dox and with or without 0.5 μM 5Z-7 were conducted. Cell colonies were stained and photographed after 14 days. (**C**–**D)** The colony numbers of HeLa (C) and C-33-A (D) cells were calculated. All values are expressed as the mean standard deviation (SD). *P* values < 0.05 (*), < 0.01 (**), or < 0.001 (***) were indicated.

To test the effect of inhibition of TAK1 in combination with Dox on anchorage-independent growth abilities, soft agar assays were performed with HPV positive HeLa and HPV negative C-33-A treated with a combination of the two drugs. Although single use of low dose 5Z-7 (0.5 μM) could not inhibit anchorage-independent cell growth, the combination with Dox could significantly strengthen the Dox-induced inhibitory effects of anchorage-independent growth on HeLa (Figure 3A) and C-33-A (Figure 3B) cells. Consistently in contrast to single Dox treatment, the combination of 5Z-7 and Dox significantly decreased colony numbers of HeLa (Figure 3C) and C-33-A cell lines (Figure 3D). These results indicate that the 5Z-7 could significantly strengthen the Dox-induced inhibitory effects of anchorage-independent growth abilities on cervical cancer cells.

### TAK1 inhibitor 5Z-7-oxozeaenol enhances Dox-induced apoptosis on cervical cancer cells

**Figure 4:**
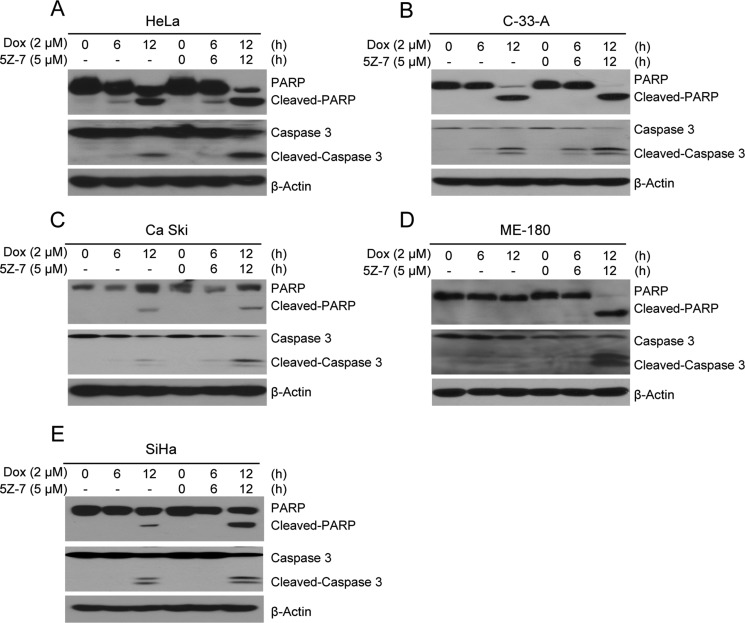
5Z-7-oxozeaenol promotes Dox-induced apoptosis (**A**–**E)** HeLa (A) and C-33-A (B), Ca Ski (C), ME-180 (D), SiHa (E) cells were treated with Dox at the indicated time points (0, 6, 12 h) with or without 5Z-7 and protein extracts were subjected to SDS-PAGE and immunoblotted for anti-PARP, and anti-Caspase 3 antibodies. β-Actin was detected as a loading control for all whole cell extracts.

Owing that single 5Z-7 treatment exhibited inhibitory efficacy on cervical cancer cells, we then tested its combinatory effects with conventional compound Dox. The results demonstrate that by strong contrast to single Dox treatment, the combination of Dox with 5Z-7 greatly increased PARP and Caspase 3 cleavages (Figure 4A–4E). These data indicate that 5Z-7 enhances Dox-induced apoptosis.

### TAK1 inhibitor 5Z-7-oxozeaenol suppresses Dox-induced NF-κB, JNK and p38 signaling

**Figure 5:**
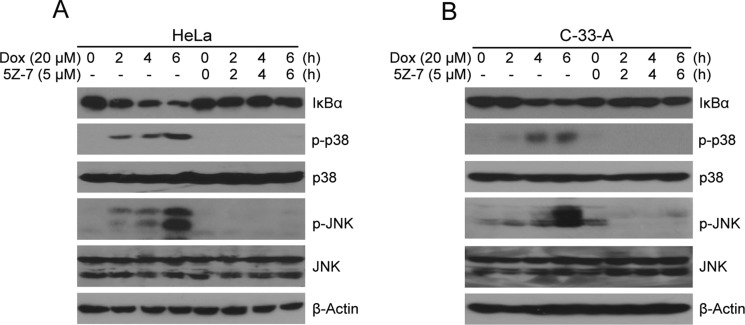
5Z-7-oxozeaenol inhibits Dox-induced NF-κB, JNK and p38 activation (**A**–**B)** HeLa (A) and C-33-A (B) cells were treated with Dox at the indicated time points (0, 2, 4, 6 h) with or without 5Z-7 and protein extracts were subjected to SDS-PAGE and immunoblotted with the indicated antibodies. β-Actin was detected as a loading control for all whole cell extracts.

NF-κB isan important transcriptional factor associated with tumor growth and cell survival [39, 40]. According to prior studies, TAK1 inhibition-mediated cell death is associated with inhibition of NF-κB and JNK/MAPK signal pathway. We therefore hypothesized that 5Z-7 would block the activation of NF-κB, JNK and p38 signaling. To investigate this hypothesis HeLa and C-33-A cells were treated with Dox or in combinations with 5Z-7. The results indicate that TAK1 inhibition by 5Z-7 significantly stabilizes IκBα and in turn suppresses NF-κB activation (Figure 5A, 5B). The treatment also successfully blocks Dox-induced JNK and p38 phosphorylation (Figure 5A, 5B). These data signify that TAK1 is required for Dox-induced pathways activation such as NF-κB, JNK and p38 in cervical cancer cells. And 5Z-7 works as a TAK1 inhibitor to block Dox induced NF-κB, JNK and p38 activation in cervical cancer cells.

## DISCUSSION

Uncovering the molecular mechanisms that mediate chemoresistance of recurrent and refractory cervical cancer patients is getting increased interest [41, 42]. NF-κB has been shown to be one of the most important transcriptional factors promoting tumor cell survival and plays an important role in cervical cancer development [39, 40]. According to prior studies, the activation of NF-κB contributes to chemotherapy resistance [11, 12], and the inhibition of NF-κB could enhance cytotoxic effects of chemotherapeutic drugs in cervical cancer [43]. Since it is clear that TAK1 plays a pivotal role in chemotherapy-induced NF-κB activation, we surmised that inhibition of TAK1 activation would block chemotherapy resistance induced by NF-κB activation and increase the efficacy of conventional chemotherapeutic agents.

5Z-7, is a novel TAK1 inhibitor that has been studied in lymphoma [38], neuroblastoma [37], fibroblasts [44] and breast cancer [45]. A closely related analog of 5Z-7, termed E6201, is in phase II clinical trials by Eisai Inc., for the treatment of melanoma, as well as being a possible topical agent for treatment of psoriasis [45]. And based on prior studies, treatment with 5Z-7 significantly enhanced the levels of reactive oxygen species (ROS) in HeLa [45], we surmise that the introduction of 5Z-7 into cervical cancer could be a potential anti-tumor strategy. Consistent with our hypothesis, our results from this *in vitro* study of a panel of cervical cancer cell lines demonstrate that inhibition of TAK1 activity greatly enhances Dox-induced cytotoxicity in cervical cancer. In this work, we used the TAK1 inhibitor 5Z-7, and found that the treatment of 5Z-7 could inhibit cervical cancer cell proliferation in dose-dependent manner (Figure 1A, 1B). And the combination of low concentration 5Z-7 with Dox showed significant inhibitory effect on the proliferation of cervical cancer cells. In addition, 5Z-7 enhanced the inhibitory effects of Dox on colony formation abilities (Figure 2) and anchorage-independent growth (Figure 3) of cervical cancer cells. In this study, we found that 5Z-7 significantly increased the cytotoxic effect of Dox (Figure 4) and inhibited Dox-induced NF-κB, JNK, and p38 activation (Figure 5). 5Z-7 is a specific TAK1 inhibitor that works by irreversibly interacting within the ATP binding site of TAK1 [46, 47]. Thus, our results suggest that TAK1 kinase activity is required for proliferation and anchorage-independent growth of cervical cancer cells. In addition, the combinations performed well by decreasing the need of higher compound dose and longer treatment time, as well as enhancing apoptosis evidenced by more PARP and Caspase 3 cleavages on cervical cancer cell line tested. These data suggest that although single treatment of low dose 5Z-7 or Dox could not fully induce cell death, however, the combination of 5Z-7 with Dox significantly enhanced Dox-induced apoptosis and fully overcome the chemoresistance.

Another important issue is the whether HPV status would influence the efficacy of TAK1 inhibitor 5Z-7. In view that persistent infection by oncogenic HPV types is a prerequisite for the development of cervical cancer, in addition, HPV16 and HPV18 are known to cause around 70% of cervical cancer cases [48]. Thus, whether HPV status would influence the effects of 5Z-7 in cervical cancer remains as a thought-provoking issue. Importantly, 5Z-7 contributes to the Dox-induced apoptosis of both HPV positive and negative cervical cancer cell lines and, thus, could be used to treat both subtypes of cervical cancer. These data pave the way for the combination of 5Z-7 with Dox to be used as a promising therapeutic strategy for both anti-HPV positive and anti-HPV negative cervical cancer.

In summary, our work shows that pharmacological inhibition of TAK1 activity blocks genotoxic stress-induced TAK1 signaling and pushes cervical cancer cells towards death pathway. Our results provide an understanding of TAK1-dependent survival pathways in response to chemotherapy and valuable data for potential clinical applications of TAK1 inhibitors like 5Z-7 to treat cervical cancer. It's the first time that we show TAK1 inhibitor 5Z-7-oxozeaenol could be used for the treatment of cervical cancer. Meanwhile, TAK1 inhibitor 5Z-7-oxozeaenol shows inhibitory effects by attenuating cell proliferation and inducing apoptosis on both HPV-positive and HPV-negative cell lines. In addition, the combination studies of 5Z-7 with chemotherapy sheds light on the process of chemoresistance of refractory cervical cancer.

## MATERIALS AND METHODS

### Cell lines and cell culture

HeLa cells were grown in DMEM (Lonza, Walkersville, MD, USA) containing 10% fetal bovine serum (FBS, SAFC Biosciences), 100 units/mL penicillin, and 100 μg/mL streptomycin. C-33-A, Ca Ski, ME-180 and SiHa cells were grown in RPMI 1640 (Cellgro) containing 10% fetal bovine serum (FBS, SAFC Biosciences), 100 units/mL penicillin, and 100 μg/mL streptomycin. Cells were cultivated in 5% CO_2_ at 37°C.

### Antibodies and reagents

Anti-JNK (9252), anti-phospho-JNK (9251), anti-IκBα (9242), anti-p38 (9212), anti-phospho-p38 (9211), and anti-PARP (9532), anti-Caspase 3 (9662) primary antibodies were obtained from Cell Signaling Technology (Danvers, MA, USA). Anti-mouse (7076) and anti-rabbit (7074) secondary antibodies were obtained from Cell Signaling Technology (Danvers, MA, USA). Anti-β-Actin (A2228) primary antibody was obtained from Sigma (Sigma-Aldrich Corp, St. Louis, MO, USA). 5Z-7-oxozeaenol (499610) was acquired from Calbiochem. Dox (D1515) was purchased from Sigma (Sigma-Aldrich Corp, St. Louis, MO, USA).

### Cell viability assay

The MTT assay were conducted using MTT (MKBH9792V) (Sigma-Aldrich, Spring, TX, USA) following the manufacturer's instructions. Briefly, the cells were seeded in 96-well plates at the density of 5 × 10^3^ cells per well. After 24 h of incubation at 37°C, cells were either allowed to grow in media alone or in media containing increasing concentrations of 5Z-7 for 72 h. Cells were then observed and photographed by optical microscope. 10 μl of MTT was added into each well, and the cells were incubated for another 2 h. Then, 50 μl of DMSO was added into each well, and the cells were incubated for another 10 min. The absorbance of each well was measured at 540 nm and plotted for the cell viability curve. The CCK-8 assay were conducted as previously described [37, 49]. Briefly, cells were plated in 96-well plates at a concentration of 1 × 10^4^ cells per well. After incubating the plate for 24 h at 37°C, cells were treated with various concentrations of Dox, 5Z-7, or a combination for the indicated duration. 72 h later, replaced medium with mixture (10 μL of Cell Counting Kit-8 (Dojindo Laboratories) solution & 190 μL DMEM or RPMI 1640 medium) and incubated for another 1 h at 37°C. The relative cell viability was quantified by measuring the absorbance at 450 nm. Each experiment was performed in triplicates.

### Cell imaging

Cervical cancer cell lines (HeLa, C-33-A, Ca Ski, ME-180 and SiHa) were seeded in 96-well plates at appropriate concentrations. Cell morphologies were observed and captured by an optical microscope after 72 h of treatment with indicated concentrations (0 μM, 2 μM, 5 μM, 10 μM) of 5Z-7.

### Colony formation assay

Cervical cancer cells were seeded in 12-well plates at 2 × 10^3^ cells per well, then treated with single Dox or the combo (Dox & 5Z-7) at indicated concentrations. After 72 h, the medium containing drug were replaced with fresh medium without drug. Two weeks later, cells were fixed and stained with methanol/crystal violet and photographed. Each experiment was performed in triplicates.

### Soft agar assay

The experiments were performed as previously described [37]. Briefly, a 5% solution of agar (214220, Difco Laboratories) was autoclaved. Followed by cooling to 56°C in a water bath. A 0.5% lower gel (mixture of agar, DMEM or RPMI 1640 containing 10% FBS) was plated in 6-well plates (2 mL per well). After this layer solidified, a 0.3% upper gel (mixture of agar, DMEM or RPMI 1640 media with 10% FBS) was made and mixed with each cell line at a concentration of 1 × 10^4^ cells per well (1.5 mL per well). Cells grew at 37°C in 5% CO_2_ for another 2 weeks (14 days) and stained with crystal violet. The colonies were photographed and counted by VersaDoc Imaging System (Bio-rad). Each experiment was performed in triplicates.

### Immunoblotting assay

Cell lysates were harvested by washing cells with ice-cold PBS twice and then lysed cells with RIPA lysis buffer (25 mM HEPES at PH 7.7, 135 mM NaCl, 1 % Triton X-100, 25 mM b-glycerophosphate, 0.1 mM sodium orthovanadate, 0.5 mM phenylmethylsulfonyl fluoride, 1 mM dithiothreitol, 10 μg/mL aprotinin, 10 μg/mL leupeptin, 1 mM Benzamidine, 20 mM disodium p-nitrophenylphosphate, and phosphatase inhibitor cocktail 2 and 3 (p5726 and p0044, Sigma)). Supernatants were collected, after centrifuging at 13,000 rpm for 15 min at 4°C. And then supernatants were boiled with loading followed by separated in SDS-PAGE (polyacrylamide gel electrophoresis). And SDS-PAGE were transferred to PVDF membranes. The PVDF membranes were blocked in TBST with 5% dry milk and then incubated with corresponding primary antibodies overnight at 4°C. The membranes were washed by TBST and incubated with corresponding secondary antibodies for 1 h at room temperature (25°C). The membranes were then visualized by the ECL-Plus Western detection system (GE Health Care, Buckinghamshire, UK) after washed by TBST for 3 times (15 min/each time). The anti-β-Actin antibodies were used as a loading control for whole cell extracts in all samples.

### Statistical analysis

Two-tailed Student's *t-test* and *ANOVA* were used to determine the statistical significance among drug treatment group and control group. Each assay was repeated at least twice, and representative results were presented. All values are expressed as the mean standard deviation (SD). *P* < 0.05 was considered statistically significant.

## Abbreviations

NF-κB: nuclear factor-kappa B
5Z-7: 5Z-7-oxozeaenol
Dox: Doxorubicin
VP-16: etoposide
HPV: human papillomavirus
MAPK: mitogen-activated protein kinase
DMSO: dimethyl sulfoxide
TGF-β: transforming growth factor-β
IκBα nuclear factor of kappa light polypeptide gene enhancer in B-cells inhibitor: alpha
IKK: IκB kinase
PARP: poly ADP ribose polymerase
